# Adaptive Neural Decoder for Prosthetic Hand Control

**DOI:** 10.3389/fnins.2021.590775

**Published:** 2021-04-08

**Authors:** Andrew E. Montgomery, John M. Allen, Sherif M. Elbasiouny

**Affiliations:** ^1^Department of Biomedical, Industrial and Human Factors Engineering, College of Engineering and Computer Science, Wright State University, Dayton, OH, United States; ^2^Department of Neuroscience, Cell Biology and Physiology, Boonshoft School of Medicine and College of Science and Mathematics, Wright State University, Dayton, OH, United States

**Keywords:** neural decoder, motor decoder, prosthetic hand, motor control, motoneuron modeling

## Abstract

The overarching goal was to resolve a major barrier to real-life prosthesis usability—the rapid degradation of prosthesis control systems, which require frequent recalibrations. Specifically, we sought to develop and test a motor decoder that provides (1) highly accurate, real-time movement response, and (2) unprecedented adaptability to dynamic changes in the amputee’s biological state, thereby supporting long-term integrity of control performance with few recalibrations. To achieve that, an adaptive motor decoder was designed to auto-switch between algorithms in real-time. The decoder detects the initial aggregate motoneuron spiking activity from the motor pool, then engages the optimal parameter settings for decoding the motoneuron spiking activity in that particular state. “Clear-box” testing of decoder performance under varied physiological conditions and post-amputation complications was conducted by comparing the movement output of a simulated prosthetic hand as driven by the decoded signal vs. as driven by the actual signal. Pearson’s correlation coefficient and Normalized Root Mean Square Error were used to quantify the accuracy of the decoder’s output. Our results show that the decoder algorithm extracted the features of the intended movement and drove the simulated prosthetic hand accurately with real-time performance (<10 ms) (Pearson’s correlation coefficient >0.98 to >0.99 and Normalized Root Mean Square Error <13–5%). Further, the decoder robustly decoded the spiking activity of multi-speed inputs, inputs generated from reversed motoneuron recruitment, and inputs reflecting substantial biological heterogeneity of motoneuron properties, also in real-time. As the amputee’s neuromodulatory state changes throughout the day and the electrical properties and ratio of slower vs. faster motoneurons shift over time post-amputation, the motor decoder presented here adapts to such changes in real-time and is thus expected to greatly enhance and extend the usability of prostheses.

## Introduction

State-of-the-art prosthetic limbs are capable of sophisticated, multi-degree-of-freedom movements that mimic many physiological motions. However, we still lack advanced control algorithms to drive those prostheses at their full potential so that they function like an amputee’s natural limb (Biddiss and Chau, 2007). Current efforts are aimed at development of more robust control schemes that can access and decode sufficiently detailed information from the patient’s remaining motor system to fully exploit the advanced capabilities of state-of-the-art prostheses. While motor decoders need to be accurate in estimating the amputee’s motor intent, they also need to be fast to support real-time operation for natural and seamless control (Cordella et al., 2016).

Two types of biological signals have been commonly used to control prosthetic limbs: electromyographic (EMG) and neural (i.e., electroneurogram, ENG) signals. In both signals, the spiking activity of spinal motoneurons (MNs) is extracted—via threshold-crossings detection methods—which motor decoders decrypt to generate a command signal to the prosthesis that is proportional to the amputee’s motor intent (Warren et al., 2016). The spiking activity of MNs contains highly detailed information on the graded activation of individual muscles (i.e., speed and direction of the intended movement) and is therefore a faithful representation of the amputee’s motor intent.

One of the greatest barriers to prosthesis usability is the rapid degradation of their control systems’ performance over time, when used in real life by amputees (Dantas et al., 2019). Thus, the user is burdened by inaccurate responses to intended movements or frequent recalibration sessions, or often both. Performance degradation occurs primarily because motor decoders are usually developed with a limited range of MN firing activities during the training phase, and because control systems are calibrated to the amputee’s current condition at the time of calibration. Yet MNs exhibit a broader variety of firing behaviors that result from different recruitment profiles of MNs, such as orderly (i.e., in which MNs are recruited from smallest to largest), mixed, or reverse recruitment, which all have been seen in animals and humans (for review, see Cope and Brian, 1995; Heckman and Enoka, 2012; Bawa et al., 2014). Also, the amputee’s neuromodulatory state (neuronal excitability level) changes throughout the day; thus the training data do not represent all possible neural states of firing activity (Dantas et al., 2019). Therefore, these factors lead to ongoing fluctuations in MNs’ firing activity, which degrade control performance over a few hours to days, requiring frequent calibration of the motor decoder to update its parameter values.

Another, more long-term problem is that post-amputation MNs frequently undergo changes in their electrical properties, changes in the proportions of MN types, and neurodegeneration (Titmus and Faber, 1990), leading to higher heterogeneity in the electrical properties of remaining MNs. These changes are ongoing well past the primary injury, and would require decoder recalibration after a longer period (months). However, in this case, the decoder might need to function with a highly variable spiking signal from MNs, and with only one or two MN types available. As a result, novel decoding algorithms are currently under development and testing to overcome these limitations (Dantas et al., 2019). A motor decoder that can auto-adapt to the more rapid dynamic changes to support long-term integrity of control systems, and also provide highly accurate, real-time movement response, would represent a major advance in prosthesis technology and amputee quality of life. Another highly desirable advance would be a decoder that only requires occasional re-training/calibration sessions to accommodate signal and MN type changes emerging after injury.

Accordingly, the first major goal of this work was to develop an adaptive MN activity-based decoder that can auto-switch between algorithms in real-time. This allows the decoder to adapt to signal changes by engaging the optimal parameter settings for decoding the MN spiking activity from the firing motor pool. Such a characteristic would reduce the need for frequent decoder calibration. The second major goal was to test the robustness of our adaptive decoder against a variety of complications that emerge after amputation. For instance, we assessed the decoder’s performance in response to post-amputation changes in the ratio of remaining MN types (slower vs. faster), signal variability resulting from increased biological heterogeneity in the MN pool, and variation in MN recruitment pattern (orderly vs. reverse recruitment), which all directly impact MN spiking activity (Titmus and Faber, 1990).

**FIGURE 1:**
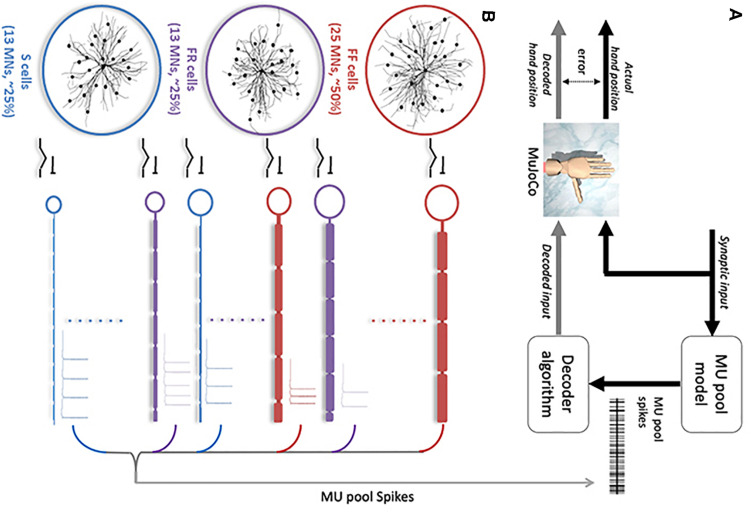
Overview of decoder development and testing paradigm. **(A)** Block diagram illustrating the steps involved in the decoder development and testing. An excitatory synaptic input is simulated in the MU pool model (shown in **B**), which generates action potentials in recruited MNs. Action potential spikes from individual MNs (MU pool spikes) serve as the input to the motor decoder. The decoder algorithm decodes the synaptic input and drives the MuJoCo prosthetic hand to move. The error between the decoded and actual hand position of the MuJoCo hand is calculated to assess the decoder performance. **(B)** Schematic diagram of the motor unit pool model published in Allen and Elbasiouny (2018) used to develop the decoder algorithm. The MU pool model consists of anatomically accurate, information of S- (blue), FR- (purple), and FF-MNs (red) with axon and soma sizes and 3D dendritic reconstructions, firing characteristics and type proportions as seen in the MG cat MN pool.

To support development and validation of this decoder as well as its testing under post-amputation conditions, we employed a multi-scale, high-fidelity computational model which represents the spinal MN pool in high detail. This model includes dendritic trees with full 3D anatomical detail, different cell types, and the ionic mechanisms that modulate neuronal excitability (Allen and Elbasiouny, 2018). This model, developed in our prior work, is based on detailed neurophysiological data and is validated to simulate MN pool firing behaviors and recruitment patterns with an unprecedented level of accuracy. To test its real-time performance in prosthetic hand control, the decoder was used to drive the MuJoCo Physics Simulator’s Luke hand prosthetic model (Todorov et al., 2012; Kumar and Todorov, 2015); then the resulting movement was compared to the movement generated by the original (pre-decoder) neural signal. The difference between the two movements was used to assess the decoder accuracy (Figure 1A). In this way, these “clear box” simulations allowed: (1) full control of all parameters in the testing environment, (2) full knowledge of the decoder‘s input and output signals, and (3) testing of the decoder with neural signals generated from different activation speeds, from specific cell types, from different MN recruitment patterns, and under conditions of biological heterogeneity in MN cellular properties. Accordingly, this approach enabled us to quantify the decoder’s performance with an exquisite level of accuracy and provide a rigorous proof of concept and performance under varied conditions expected to arise after amputation.

Our results show that an adaptive decoder algorithm based on the MN pool spiking rate operated with a high level of accuracy [Pearson’s correlation coefficient > 0.99 and Normalized Root Mean Square Error (NRMSE) ∼5%] and in real-time (decoding time < 10 ms), while adapting to a wide range of physiological conditions. Although calibrated to spiking activity resulting from fixed-speed inputs of orderly recruited MNs, the decoder robustly decoded the spiking activity of multi-speed inputs, inputs generated from reversed MN recruitment, and inputs involving substantially greater biological heterogeneity of MN properties (Pearson’s correlation coefficient > 0.98 and NRMSE <13%). Accordingly, these results support the feasibility of the present adaptive decoder for prosthetic control and for adapting to expected post-amputation changes.

## Materials and Methods

### Computational Model

**FIGURE 2:**
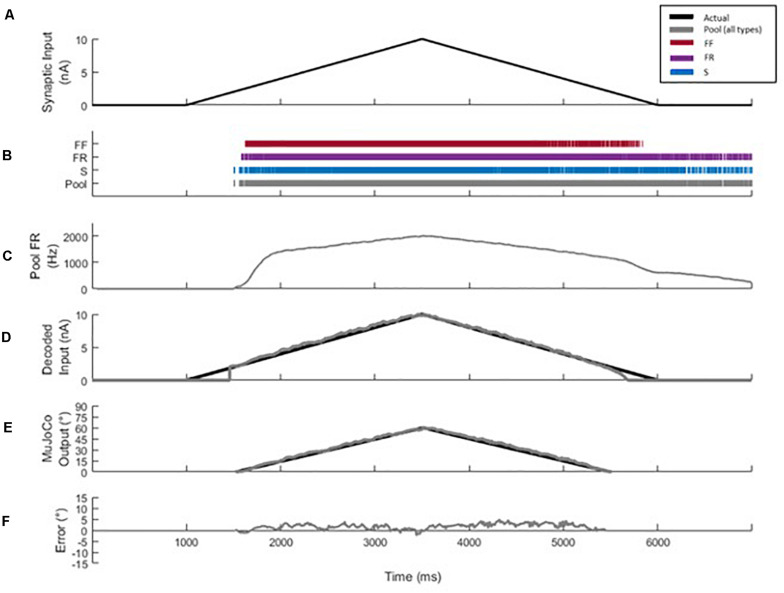
Decoder development and performance. **(A)** Profile of the excitatory synaptic input command to the pool model to activate the motor pool. The input increases and decreases at 4 nA/s to a peak level of 10 nA. **(B)** Spikes of type S (blue), FR (purple), and FF (red) MNs are combined to form the aggregate motor pool spikes (gray). The spikes highlight the recruitment order and self-sustained firing characteristics of S and FR cell types, relative to the FF type. **(C)** Smoothed aggregate firing rate of the motor pool. **(D)** Comparison of decoded vs. actual synaptic inputs. **(E)** Profiles of MuJoCo’s hand position when driven by the actual synaptic input (black) vs. the decoded input (gray). **(F)** The instantaneous error in MuJoCo’s hand position, calculated as the difference between MuJoCo’s hand position angles when driven by the actual vs. the decoded inputs (same color code as in D).

The 51-MN homogeneous pool model described in Allen and Elbasiouny (2018) was activated via synaptic inputs (Figure 1B) at a rate of 4 nA/s to a peak level of effective synaptic current of 10 nA, after which the level of activation was reduced at a rate of −4 nA/s until the measured input at the somas returned to 0 nA (Figure 2A). This input profile is of sufficient magnitude to recruit 100% of S and FR-type MNs and 80% of FF-type MNs, which constitutes 90% recruitment of the full pool (activation level = 90%). The aggregate spiking (Figure 2B, gray) as well as spiking of each cell-type population (Figure 2B, red, purple, and blue traces) were recorded during the simulations. The firing rate of the aggregate pool spiking activity was calculated by the decoder (Figure 2C); then the decoder’s performance in decoding the pool spiking activity was assessed by comparing MuJoCo’s movement driven by the actual input vs. the decoded input (Figure 2E, black vs. gray trace). The difference between the two movements were calculated as the instantaneous error (Figure 2F). Part of this work appeared in Montgomery (2018).

### Collection and Processing of Spiking Data

The synaptic conductances inserted along the dendrites of MNs in the MU pool model were adjusted to simulate an input stimulus to the pool, and the somatic membrane potential of each MN was recorded. Action potential (AP) spikes of MNs were identified, counted, and averaged via scrolling 50 ms bins with 10 ms update intervals (i.e., the first 40 ms of the new bin is comprised of the last 40 ms of the previous bin). In that way, the 10ms update interval in each bin contains the most recent pool spiking data. This scrolling average feature provided the smoothing effects of a 50 ms bin average, but with only a 10 ms latency.

### Brown’s Linear Exponential Smoothing

Brown’s method (Hansun, 2016) for exponential smoothing was applied to reduce the variation in spike count between successive time bins. The smoothed spike count *y*_*t*_ is modeled by a linear regression equation as the sum of intercept *b*_0_ and slope *b*_1_ which are estimates of the spike count’s level and trend, respectively.

(1) $$y_{t}=b_{0}+b_{1}$$

Estimates of *b*_0_ and *b*_1_ are calculated by maintaining two exponentially smoothed parameters $x_{t}^{\prime }$ and $x_{t}^{\prime \prime }$.

(2) $$b_{0}=2x_{t}^{\prime }-x_{t}^{\prime \prime }$$

(3) $$b_{1}=\frac{\alpha }{\left(1-\alpha \right)}\left(x_{t}^{\prime }-x_{t}^{\prime \prime }\right)$$

The first exponentially smoothed parameter $x_{t}^{\prime }$ is a weighted sum of the present spike count *x*_*t*_ and the previous bin’s smoothed parameter *x*′*_*t*_*_–__1_. The second exponentially smoothed parameter $x_{t}^{\prime \prime }$ is the weighted sum of the present first exponentially smoothed parameter *x*′*_*t*_* and the previous bin’s second exponentially smoothed parameter *x*″*_*t*_*_–__1_.

(4) $$x_{t}^{\prime }=\alpha x_{t}+\left(1-\alpha \right)x_{t-1}^{\prime }$$

(5) $$x{"}_{t}=\alpha x_{t}^{\prime }+\left(1-\alpha \right)x_{t-1}^{\prime \prime }$$

These parameters’ recursive origins allow the single weighting parameter (*α* = 0.08) to apply an exponentially decreasing weight to past spike count observations. This results in a smoothed value that is the weighted average of the most recent spike count bin and many previous spike count bins while avoiding the computational expense of maintaining a vector of saved previous spike count observations. The smoothed spike count and fixed bin size of 50 ms are then used to convert the spiking activity to a firing rate value in Hertz.

### Decoder Calibration and Auto-Switching

Spinal MNs respond to a linear synaptic activation with frequency-current (FI) relationships of two firing phases of different gains (i.e., secondary and tertiary firing phases/gains). These different phases and gains result from the graded activation of persistent inward currents (PICs), with partial PIC activation and low firing gain during the secondary firing phase, but full PIC activation and high firing gain during the tertiary firing phase (Lee et al., 2003). Accordingly, the decoder was developed to account for this non-linearity in the MN spiking behavior and to expect the MU pool’s firing gain to change in two phases in response to synaptic input activation. Thus, synaptic input to the pool was estimated according to the following equation:

(6) $$SynapticInput\left(nA\right)=RTE+\frac{y_{t}-MFR}{g}$$

(7) $$g=\frac{\Delta FiringRate}{\Delta SynapticInput}$$

in which *g* is the gain of the linear FI relationship, RTE is the recruitment threshold excitation or the minimum synaptic input needed for the onset of firing, MFR is the minimum firing rate at onset of firing, and *y*_*t*_ is the current smoothed firing rate (see section “Brown’s Linear Exponential Smoothing”).

Equation (6) was based on the work of Fuglevand et al. (1993) but modified to solve for the synaptic input of the motor pool. These parameters (RTE, MFR, and *g*) are estimated during calibration by assessing the aggregate firing output response of the pool to a triangular ramp input (Figure 2C). The decoder selects the most optimal RTE, MFR, and *g* from these calibrated values based on the firing rate of the most recent time bin. The decoder uses the rate of change of the smoothed firing rate (*y′_*t*_*) to switch between the settings and engage the most optimal values of RTE, MFR, and *g*. A high rate of change corresponds to the secondary firing phase and fast activation, while a low rate of change corresponds to tertiary firing and slower activation.

The decoder was calibrated using current ramp activation rates of 2, 4, and 6 nA/s. Each firing phase is calibrated with a particular portion of the firing response. The secondary phase parameters are set relative to the onset and initial acceleration in pool firing, corresponding to aggregate firing occurring from 1,500 to 2,000 ms in Figure 2C. The region of the firing response where this acceleration is seen to decrease while the activation rate of the ramp remains constant is treated as the pool’s transition to tertiary firing. Calibration of the tertiary phase is then set using the firing response from this transition region to the peak level of firing/activation, aggregate firing occurring from 2,000 to 3,500 ms in Figure 2C.

### Decoder Testing and Performance Assessment

The decoder’s performance was tested and assessed by comparing the resultant movement of the MuJoCo Physics Simulator’s Luke hand prosthetic model (Todorov et al., 2012; Kumar and Todorov, 2015) when driven by the actual input to the pool vs. driven by the decoder’s output (Figure 2E). A conversion scheme was established to relate the input magnitude in nano-amperes (nA) to the prosthetic model’s finger position angle (degrees). The peak synaptic input of 10 nA corresponds to 60° of finger flexion, with all other values from zero to the peak maintaining a linear relationship, given the linearity of the input stimulus. In order to obtain the movement which would result from the actual input, the true value of synaptically injected current for the given time instance of simulation was used to drive MuJoCo directly, and the resulting position was recorded. This vector then serves as the reference for comparison of any subsequent decoding efforts of firing output in response to the particular synaptic input profile.

The decoder’s performance was assessed using two primary metrics:

(1) *Pearson’s linear correlation coefficient* (Pearson’s CC, Lee Rodgers and Nicewander, 1988; Artemiadis and Kyriakopoulos, 2007): The Pearson’s CC was used to measure the correlation between MuJoCo’s hand positions when driven by the actual vs. decoded inputs. Pearson’s CC was measured by the following equation:
  (8) $$CC=\frac{\sum_{k=1}^{N}\left(z_{k}-\bar{z}\right)\left({\hat{z}}_{k}-\bar{\hat{z}}\right)}{\sqrt{\sum_{k=1}^{N}{\left(z_{k}-\bar{z}\right)}^{2}\sum_{k=1}^{N}{\left({\hat{z}}_{k}-\bar{\hat{z}}\right)}^{2}}}$$
  where *z*_*k*_ and $\bar{z}$ are the instantaneous and mean MuJoCo’s hand positions when driven by the actual input, respectively, and ${\hat{z}}_{k}$ and $\bar{\hat{z}}$ are the instantaneous and mean MuJoCo’s hand positions when driven by the decoded input, respectively.
(2) *Normalized Root Mean Square Error* (NRMSE, Sheiner and Beal, 1981; Artemiadis and Kyriakopoulos, 2007): The NRMSE, expressed as a percentage, was used to measure the error between MuJoCo’s hand positions when driven by actual vs. decoded inputs, and was measured by the following equations:
  (9) $$RMSE=\sqrt{\frac{1}{N}\sum_{k=1}^{N}{\left({\hat{z}}_{k}-z_{k}\right)}^{2}}$$
  (10) $$NRMSE\left(\%\right)=\frac{RMSE}{\max\left(z_{k}\right)-\min\left(z_{k}\right)}$$
(3) *Root Mean Square Error (RMSE):* While NRMSE is widely used to assess decoders performance, it shows the RMSE error value normalized to the difference between min and max values, not its absolute value. To provide the actual error value, we also report the RMSE value (calculated in Equation 9) for each condition.

Together, the Pearson’s CC, NRMSE, and RMSE metrics provide a comprehensive assessment of the quality of the decoder’s estimates. The datasets presented in this study are available as Supplementary Material.

## Results

### Development Stage and Computational Platform

Our first goal was to develop a motor unit firing activity-based decoder capable of translating neural signals (decoder input) in real-time into an accurate motor command (decoder output/prosthesis input). To this end, we employed a multi-scale, high-fidelity motor unit (MU) pool model developed by Allen and Elbasiouny (2018) as a computational platform to aid in decoder development (see section “Discussion” for details). This model incorporates the nonlinearities involved in ion channel activation and the generation of MN spikes from synaptic inputs.

Figure 1A shows a block diagram that illustrates the general steps involved in the motor decoder algorithm development and testing, while Figure 1B shows detail of the MU computational model used to generate the MU pool spikes. The MU pool model includes cellular models of S, FR, and FF motoneuron (MN) types whose electrical and membrane properties have been optimized and rigorously verified to simulate the firing behaviors of spinal MNs (Allen and Elbasiouny, 2018). Synaptic conductances on the dendrites of each MN in the pool model were varied linearly in order to provide a triangular synaptic input to each cell (Figures 1B, 2A). When activated, cells fire action potentials along their myelinated axons; then potentials combine from all cells to form a train of pool spikes (Figure 2B) from which the instantaneous pool firing rate is calculated (Figure 2C). This train of pool spikes served as the input to the motor decoder shown in Figure 1A.

### Decoder Development, Calibration, and Testing

The objective of the motor decoder algorithm is to estimate the magnitude of the synaptic input (i.e., excitation level) to the MNs from the train of pool spikes (decoder input). This estimated excitation level (decoder output) was used to drive the opening and closing movements of the simulated MuJoCo prosthetic hand (i.e., prosthesis input) (Todorov et al., 2012; Kumar and Todorov, 2015). The train of pool spikes (Figure 2B) was smoothed (see section “Materials and Methods” for detail) and input into the decoder.

When tested in response to a triangular synaptic input, the motor decoder demonstrated high performance in estimating the excitation level to the MU pool as shown in Figures 2D–F (compare the black to gray traces in Figures 2D–F). Specifically, the decoded synaptic input matched closely the actual synaptic input with NRMSE of 3.97%, RMSE of 0.3971, and Pearson’s CC of 0.9948 (Figure 2D). To assess the functional performance of the motor decoder, we also compared MuJoCo’s hand movement when driven by the decoded synaptic current vs. when driven by the actual synaptic input (Figure 2E). MuJoCo’s hand position was very comparable when driven by decoded vs. actual synaptic inputs (compare black to gray traces in Figure 2E), with NRMSE of 3.58%, RMSE of 2.14, and Pearson’s CC of 0.9972. Because the profile of the decoded synaptic input current is similar to that of MuJoCo’s movement, we will only present MuJoCo’s hand position in the rest of the figures. The instantaneous error in MuJoCo’s hand movement, assessed as the difference between MuJoCo’s decoded and actual hand positions, was insignificant (Figure 2F). Collectively, these results show that a motor decoder algorithm that is based on the motor pool firing activity is capable of driving accurate prosthetic hand movement.

### Cell Type Testing

**FIGURE 3:**
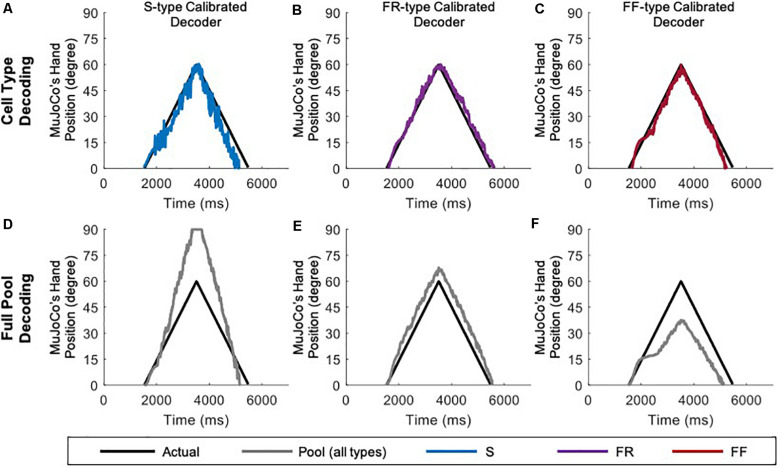
Performance of cell-type calibrated decoders in response to a triangular ramp with rate of rise and fall of 4 nA/s. S-type calibrated decoder tested on the firing of S MNs only **(A)** and on aggregate pool firing **(D)**. FR-type calibrated decoder tested on the firing of FR MNs only **(B)** and on aggregate pool firing **(E)**. FF-type calibrated decoder tested on the firing of FF MNs only **(C)** and on aggregate pool firing **(F)**.

Given that transition in MU types (S-to-FR, FR-to-FF, etc.), and therefore shift in their ratios, is seen in different muscles after amputation (Titmus and Faber, 1990), we examined whether the firing activity of one cell type is a better predictor of the pool excitation level than other cell types. To achieve that, we compared the motor decoder’s performance in driving the MuJoCo hand when calibrated to the firing activity of each cell type separately, but tested on the aggregate pool firing activity (Figure 3). For instance, an S-type-calibrated decoder estimated the synaptic input of S cells reasonably (see Table 1 for NRMSE, RMSE, and Pearson’s CC) but with some degree of underestimation (Figure 3A). Because S-MNs have high input resistance and strong PICs, they experience a greater initial increase in firing gain at recruitment (i.e., the secondary range). However, once their PICs have saturated, S-MNs fire at lower firing gain (i.e., the tertiary range), which underestimates the magnitude of synaptic input later on and leads to reduced MuJoCo hand movement throughout the rest of the ascending and all of the descending phases (Figure 3A). However, when tested on the aggregate pool firing activity, the S-type calibrated decoder significantly overestimated the synaptic input of the pool, indicated by exaggerated MuJoCo hand movement relative to actual synaptic input (Figure 3D), with high NRMSE and RMSE values (Table 1). This overestimation is due to the decoder parameters, which have been calibrated to the S-MNs’ low firing activity, causing the decoder to expect modest increases in firing activity to continued synaptic input increases. Given that the firing rate of the pool firing activity is higher than that of S-MNs, this leads to overestimation of the decoded synaptic input (Figure 3D).

**TABLE 1:** Cell-type neural decoders’ performance measures.

Calibration condition	Test condition	MuJoCo’s hand position		
		NRMSE	RMSE	Pearson’s CC
S-type	S-type firing	9.90%	6.0597	0.967337
	Pool firing	16.53%	19.635	0.983047
FR-type	FR-type firing	5.12%	3.0733	0.993292
	Pool firing	6.43%	7.7266	0.993930
FF-type	FF-type firing	5.47%	3.2006	0.989249
	Pool firing	29.29%	14.454	0.993241

The opposite was true for an FF-type calibrated decoder, which estimated the synaptic input of FF-MNs well (Figure 3C, see Table 1 for NRMSE, RMSE, and Pearson’s CC values), but severely underestimated the synaptic input of the pool, indicated by reduced MuJoCo hand movement relative to actual synaptic input (Figure 3F). This underestimation is due to the decoder parameters, which have been calibrated to the FF-MNs’ high firing activity, causing the decoder to expect high firing activity to continued synaptic input increases. Given that the rate of the pool firing activity is lower than that of FF-MNs, this leads to underestimation of the decoded synaptic input (Figure 3F). Because of their intermediate firing activity, an FR-type calibrated decoder demonstrated the best performance (with the smallest NRMSE and RMSE values and highest Pearson’s CC values among the other cell types, Table 1), estimating the synaptic input of FR-MNs as well as the excitation level to the MN pool reasonably (Figures 3B,E). In sum, these results show that the firing activity of FR-MNs is a better predictor of the magnitude of synaptic input to the MN pool than are S- or FF-MNs. These results are expected to be informative to decoder recalibration to accommodate neurodegeneration and/or shifts in available MN types that occur after the primary injury.

### Single-Speed Motor Decoder

**FIGURE 4:**
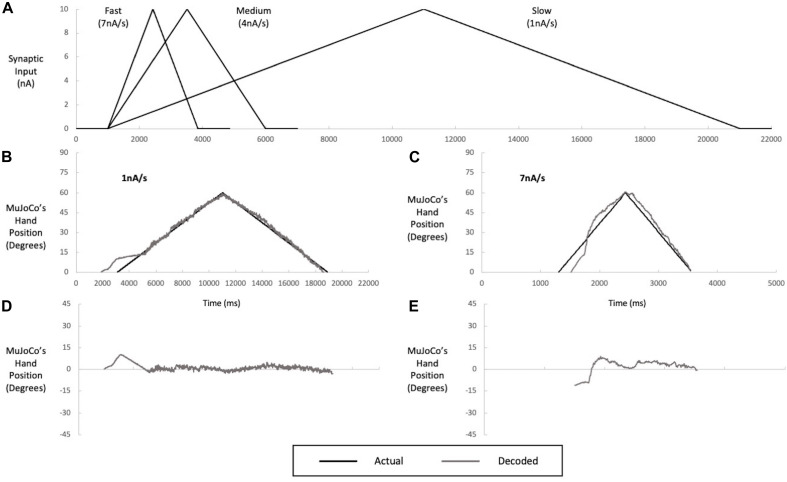
Multi speed testing of a single-speed decoder (calibrated to an intermediate speed input of 4 nA/s). **(A)** Inputs of slow (1 nA/s), medium (4 nA/s), and fast (7 nA/s) speeds used in decoder calibration and testing. The decoder was calibrated to the medium speed but tested on the slow **(B)** and fast **(C)** speeds. Performance of the medium-speed calibrated decoder when tested with slow **(B)** and fast **(C)** inputs, illustrated by a comparison of MuJoCo’s hand position when driven by the actual (black) vs. decoded (gray) inputs. The instantaneous error in MuJoCo’s hand position when the medium-speed calibrated decoder was tested with slow **(D)** and fast **(E)** Inputs.

Decoders need to be able to drive a prosthesis at different speeds, so we next examined whether a motor decoder algorithm calibrated to synaptic input of a single speed would function with equal accuracy at other speeds. We therefore tested the single-speed motor decoder presented above (calibrated to the pool’s aggregate firing activity at activation speed of 4 nA/s) with synaptic inputs of two other activation speeds: 1 nA/s (a slow activation speed, close-to-open hand movement of 12 s) and 7 nA/s (a fast activation speed, close-to-open hand movement of 1 s) (Figure 4A). When the MU pool model was activated at 1 nA/s, the firing of the most excitable S-MNs started earlier, by ∼400 ms, at a lower input level than when activated at the calibration speed of 4 nA/s. The early recruitment of MNs is due to the higher effectiveness of slow synaptic inputs in activating the dendritic PICs, leading to earlier cell recruitment. This change in firing onset resulted in earlier MuJoCo movement onset than that evoked by the actual input (Figure 4B). Other than that, the decoder’s performance was excellent for the rest of the ascending and descending phases (Figure 4B), as indicated by the small error in MuJoCo movement (Figure 4D). The NRMSE, RMSE, and Pearson’s CC values are shown in Table 2.

**TABLE 2:** Summary of performance measures for the different types of neural decoders.

Decoder type	Calibration speeds	Test speeds	MuJoCo’s hand position		
			NRMSE	RMSE	Pearson’s CC
Single-speed decoder	4 nA	1 nA	4.67%	2.4662	0.9917
		4 nA	3.58%	2.1402	0.9973
		7 nA	8.74%	5.1711	0.9731
Two-speed decoder	2 nA	1 nA	4.61%	2.7656	0.9962
		2 nA	5.28%	3.1583	0.9945
		3.5 nA	7.01%	4.1752	0.9838
	6 nA	3.5 nA	5.89%	3.5202	0.9782
		6 nA	3.72%	2.2115	0.9928
		7 nA	5.30%	2.9196	0.9925
Three-speed decoder	2 nA	1 nA	4.61%	2.7737	0.9962
		2 nA	5.28%	3.1497	0.9944
		3 nA	6.30%	3.6940	0.9898
	4 nA	3 nA	3.99%	2.3683	0.9960
		4 nA	3.58%	2.1402	0.9973
		5 nA	4.70%	2.8061	0.9936
	6 nA	5 nA	4.42%	2.6846	0.9878
		6 nA	3.72%	2.2162	0.9928
		7 nA	5.31%	5.2556	0.9925
	Orderly recruitment	2—6 nA (adaptive)	5.58%	3.3591	0.9951
	Reverse recruitment	2–6 nA (adaptive)	13.24%	9.4953	0.9854

When the MU pool model was activated with a faster input of 7 nA/s, opposing effects were observed. First, the onset of pool firing occurred at a recruitment threshold higher than that seen for the calibration speed, leading to delayed MuJoCo hand movement, by ∼200 ms as compared to that evoked by the actual input (Figure 4C). The delayed recruitment of MNs was due to the lower effectiveness of fast synaptic inputs in activating the dendritic PICs, which have slow dynamics, leading to later cell recruitment. Second, the weak PIC activation by the fast synaptic input caused low pool firing activity that persisted for some time and resulted in underestimation of the magnitude of decoded synaptic input during the first half of the ascending phase (Figure 4C). After full PIC activation, the motor decoder algorithm estimated the synaptic input reasonably with much less error (Figure 4E, NRMSE, RMSE, and Pearson’s CC values are listed in Table 2). In sum, these results demonstrate that the good performance of a single-speed motor decoder algorithm at the calibration speed does not extend well to other speeds.

### Adaptive 2-Speed Motor Decoder

**FIGURE 5:**
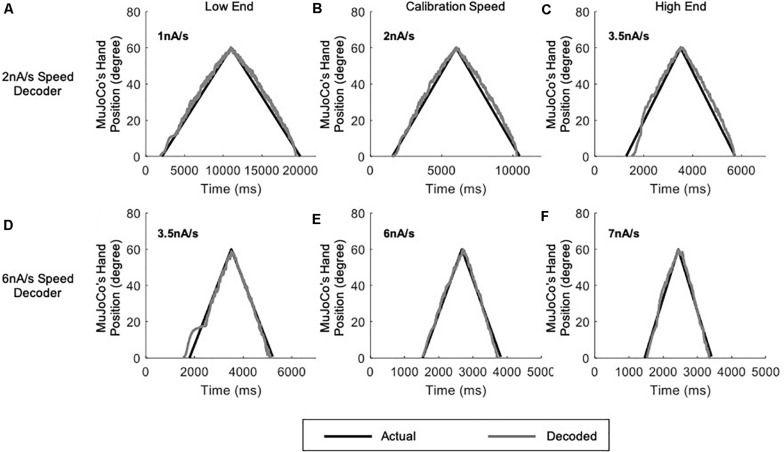
Testing of the adaptive 2-speed decoder across a range of activation speeds. All panels include comparison of MuJoCo’s hand position when driven by the actual (black) vs. decoded (gray) inputs. Decoder calibrated to 2 nA/s input tested on a 1 nA/s **(A)**, 2 nA/s **(B)**, and 3.5 nA/s **(C)** inputs. Decoder calibrated to 6 nA/s input tested on a 3.5 nA/s **(D)**, 6 nA/s **(E)**, and 7 nA/s **(F)** inputs.

In an effort to achieve optimal motor decoder performance throughout the activation speed range, we first expanded the single-speed motor decoder described above into an adaptive 2-speed motor decoder. This decoder automatically switches in real-time between two parameter settings, each calibrated to a single speed, depending on the detected pool activation speed (Figure 5). The activation speed range of the prosthetic hand (1–7 nA/s) was thus split into two sub-ranges covered by two decoder settings: One calibrated to an intermediate speed of 2 nA/s to cover the < 3.5 nA/s sub-range (Figures 5A–C), and another calibrated to an intermediate speed of 6 nA/s to cover the > 3.5 nA/s sub-range (Figures 5D–F). In this way, each decoder settings covers a small speed range around its calibration speed.

Although some errors still resulted (NRMSE, RMSE, and Pearson’s CC values are shown in Table 2), the adaptive 2-speed decoder showed much more accurate decoding performance than the single-speed decoder. Specifically, the 2 nA/s (i.e., the low-speed settings) decoded the low-end speed of 1 nA well with low error (Figure 5A and Table 2), whereas it showed higher error in decoding the high-end speed of 3.5 nA (Figure 5C and Table 2). Conversely, the 6 nA/s (i.e., the high-speed settings) decoded the high-end speed of 7 nA well (Figure 5F and Table 2), whereas it showed higher error in decoding the low-end speed of 3.5 nA (Figure 5D and Table 2). Collectively, these results indicate that our motor decoder algorithm does not perform well when tested with activation speeds > 1 nA/s away from its calibration speeds.

### Adaptive 3-Speed Motor Decoder

**FIGURE 6:**
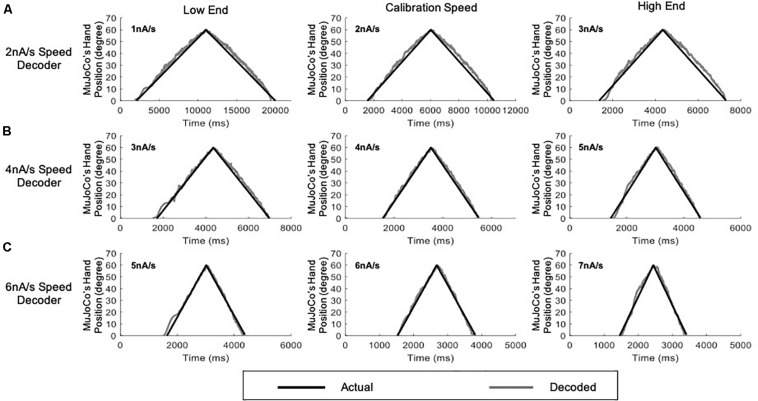
Testing of the adaptive 3-speed decoder across a range of activation speeds. All figures include comparison of MuJoCo’s hand position when driven by the actual (black) vs. decoded (gray) inputs. **(A)** Decoder calibrated to 2 nA/s input tested on a 1 nA/s (low-end speed, left panel), 2 nA/s (calibration speed, middle panel), and 3 nA/s (high-end speed, right panel) inputs. **(B)** Decoder calibrated to 4 nA/s input tested on a 3 nA/s (low-end speed, left panel), 4 nA/s (calibration speed, middle panel), and 5 nA/s (high-end speed, right panel) inputs. **(C)** Decoder calibrated to 6 nA/s input tested on a 5 nA/s (low-end speed, left panel), 6 nA/s (calibration speed, middle panel), and 7 nA/s (high-end speed, right panel) inputs.

Based on the results above, we further expanded our motor decoder framework to split the activation speed range of the prosthetic hand into three sub-ranges, each decoded by a single algorithm setting calibrated to a single speed (Figure 6). In this version, a low-speed setting algorithm calibrated to an intermediate speed of 2 nA/s covered the activation sub-range between 1 and 3 nA/s (Figure 6A). A middle-speed setting algorithm calibrated to an intermediate speed of 4 nA/s covered the activation sub-range between 3 and 5 nA/s (Figure 6B). Finally, a high-speed setting algorithm calibrated to an intermediate speed of 6 nA/s covered the activation sub-range between 5 and 7 nA/s (Figure 6C). In this way, each decoder setting covers an activation speed no more than 1 nA/s away from its calibration speed on either end. As above, the decoder automatically detected the pool activation speed and switched in real-time to the optimal parameter settings. Results of the adaptive 3-speed decoder showed greatly improved decoding performance at all testing speeds (see Table 2 for the NRMSE, RMSE, and Pearson’s CC values at each speed), as compared to the single-speed and 2-speed motor decoders presented above. Notably, the auto-switching among the pre-calibrated settings was highly efficient and the adaptive 3-speed decoder had decoding time of 8.94 ms. This time indicates that all computations are able to take place within the decoder’s inherent latency period—which is 10 ms—supporting real-time operation.

### Variable-Speed Input Testing

**FIGURE 7:**
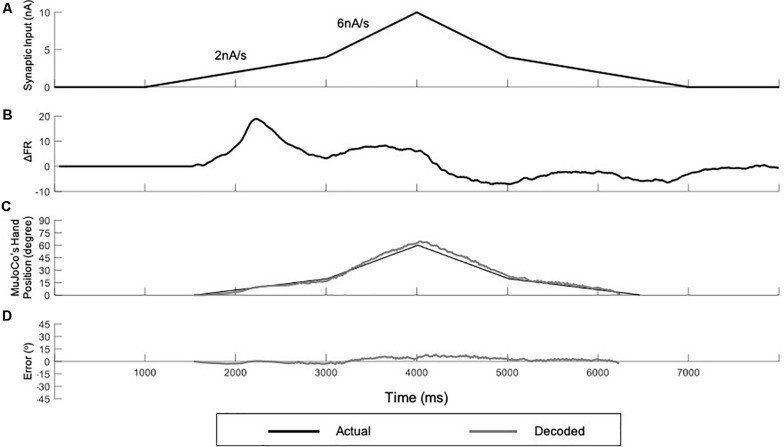
Testing of the adaptive 3-speed decoder using a variable input of changing activation speed. **(A)** Profile of the synaptic input to the pool model. The input increases at 2 nA/s for 2,000 ms, then increases by 6 nA/s for 1,000 ms to reach a peak activation of 10 nA. The input decreases at the same rates to return to zero. **(B)** The rate of change in pool firing rate. This measure is used to control the auto-switching among speed settings in the adaptive decoder. **(C)** Comparison of MuJoCo’s hand position when driven by the actual (black) vs. decoded (gray) inputs. **(D)** Instantaneous error in MuJoCo’s hand position when driven by the adaptive 3-speed decoder.

To further examine the developed decoder with inputs that reflect a more practical prosthetic use, we tested the adaptive 3-speed decoder with a variable input of changing activation speed (Figure 7). This mimicked a situation in which the amputee changes the activation speed midway while opening and closing the prosthetic hand. Under these conditions, the adaptive decoder should auto-switch in real time as it detects the changing activation speed. Figure 7A shows a variable synaptic input that was used to activate the MU pool model. The synaptic input started increasing during the hand opening phase at low speed (2 nA/s) then switched midway to a higher speed (6 nA/s). It then decreased speed back to 2 nA/s during the hand closing phase, resulting in a dual-speed ramp (Figure 7A). Figure 7B shows the rate of change in pool firing rate that was used to estimate the activation speed (i.e., from the ΔFR value) and its direction (i.e., +ve for increasing input and -ve for decreasing inputs). Although the variable input made a big jump in speed (from the lowest to the highest activation sub-ranges skipping the intermediate sub-range), the decoder still auto-switched dynamically and correctly estimated the activation speed in real-time. Note that the decoder was able to decode the variable speed input accurately on both the ascending and descending phases of input and replicated the dual-speed ramp (Figure 7C, NRMSE of 5.58%, RMSE of 3.3591, and Pearson’s CC of 0.9951) with small instantaneous error in MuJoCo position (Figure 7D). The highest error in MuJoCo hand movement did not exceed 10**°**. Additionally, as shown in Supplementary Video 1, the prosthetic hand changed its speed smoothly while opening and closing. In conclusion, these results show that the adaptive 3-speed decoder is capable of auto-switching and decoding dynamic inputs of varying activation speeds accurately in real-time.

### Biological Heterogeneity Testing

**FIGURE 8:**
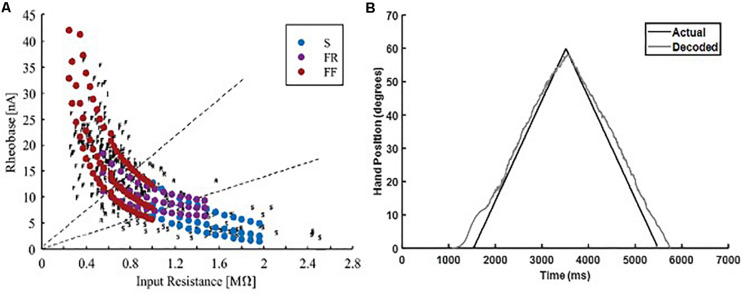
Testing of the adaptive 3-speed decoder with the firing output of a heterogeneous MN pool. **(A)** Comparison of physiological activation characteristics to activation characteristics of heterogeneous MN pool (figure adapted from Allen and Elbasiouny, 2018, with permission). **(B)** Performance of the adaptive 3-speed decoder in driving the MuJoCo prosthetic hand in response to the output of heterogeneous MU pool.

Biological heterogeneity in the electrical properties of individual MNs expands the recruitment range of MNs within each sub-type, which causes high variability in the aggregate pool spikes (Allen and Elbasiouny, 2018). Therefore, to further test the robustness of the adaptive 3-speed decoder in decoding neural activity with high firing variability, we used a heterogeneous MU pool model developed by Allen and Elbasiouny (2018) to generate MN firing activity in response to an increasing and decreasing ramp input. This heterogeneous MU pool model has a larger MN pool (153 MNs, as opposed to 51 MNs in the standard MU pool used in decoder development and calibration) and largely mimics the variability in MN cellular properties observed experimentally (Figure 8A). Such biological variability has a strong impact on the firing behaviors of MU pools (Allen and Elbasiouny, 2018), and would be expected to be encountered in human subjects. The adaptive 3-speed decoder was then tested with spike trains from the heterogeneous pool and MuJoCo’s hand movement was recorded. Figure 8B shows that the adaptive 3-speed decoder was able to decode the neural activity of the heterogeneous MU pool model reasonably (NRMSE of 6.55%, RMSE of 3.903, and Pearson’s CC of 0.9964) despite the large variability in pool spikes and firing rates. Most of the error was observed at the onset and offset of pool firing due to the expanded recruitment and de-recruitment ranges. The error decreased as a greater percentage of cells activated, and the overall direction and degree of hand movement were well predicted. Accordingly, these results demonstrate that this adaptive decoder could decode neural activity with firing variability much higher than what the decoder has been calibrated to.

### Reverse Recruitment Testing

**FIGURE 9:**
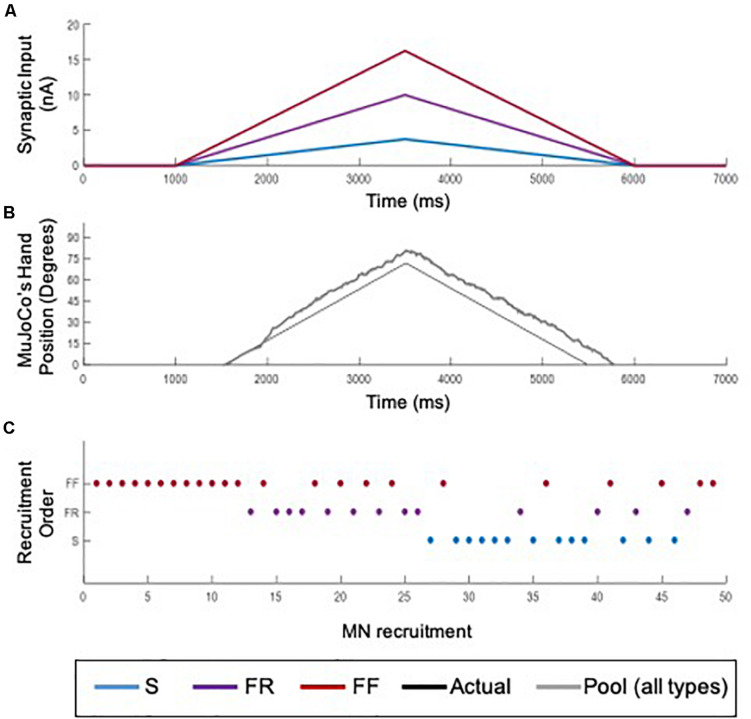
Testing of the adaptive 3-speed decoder with the firing output of a reversely recruited MN pool. **(A)** The synaptic input to MNs in the pool model. A non-uniform input activated S-MNs to a peak value of 3.75 nA (blue), FR-MNs to a peak value of 10 nA (purple), and FF-MNs to a peak value of 16.25 nA (red). **(B)** Recruitment order of MNs in the pool. The non-uniform input recruited MNs opposite to the size principle: FF-MNs first, followed by FR, then S-MNs. **(C)** Comparison of MuJoCo’s hand position when driven by the actual input (black) vs. the input decoded from the aggregate firing of reverse recruitment pool (gray).

Because different recruitment orders of MNs underlie different movements (for review, see Cope and Brian, 1995; Heckman and Enoka, 2012; Bawa et al., 2014), we examined the performance of the adaptive 3-speed decoder, which was developed and calibrated in simulations of orderly recruited MNs, in a situation of reversed recruitment. Reverse recruitment was produced by activating the MN pool model with a non-uniform synaptic input of higher magnitude to FF-type cells > FR-type > S-type cells (Figure 9A), which activated FF, then FR, then S MNs (Figure 9B). The decoder’s performance in driving the MuJoCo prosthetic hand is shown in Figure 9C. MuJoCo’s hand movement in response to the decoded input was initially very close to that of the actual input before it shifted upwards and leveled out throughout the rest of movement. This shift results from the later recruitment of S-MNs and the activation of their PICs, leading to high firing activity which sends the decoder into higher speed parameters to generate this shift. Despite that shift, the adaptive decoder was able to predict the slope and direction of the intended movement accurately and drove the MuJoCo prosthetic hand reasonably well and similar to the actual input (NRMSE of 13.24%, RMSE of 9.4953, and Pearson’s CC of 0.9854). Therefore, these results show that the developed adaptive decoder is versatile, in that it could decode the firing activity of MNs recruited in different orders (i.e., orderly or reversely recruited). This decoder’s robust performance in scenarios of realistic biological variability (3.8, above) and in non-orderly MN recruitment is expected to support accurate control in context of rapid fluctuations in the amputee’s physiological state.

## Discussion

This study sought to address, first, a major barrier to the real-life use of prostheses by amputees: the degradation of control systems’ accuracy over time, thus necessitating frequent recalibrations. Also important were the goals of improving the accuracy of detail on intended movements and real-time responsiveness. We thus developed, optimized, and tested an adaptive motor decoder algorithm that decoded the spiking activity of MNs to drive a prosthetic hand with real-time performance. As opposed to traditional approaches in which a standard algorithm (e.g., Kalman filter, deep learning decoders, etc.) is employed to learn the spiking data, the development and optimization of this decoder algorithm were guided and informed by the neurophysiological properties of spinal MNs. Furthermore, its testing was conducted in a robust simulation environment that incorporated the nonlinearities of MN spiking dynamics and recruitment. This approach, therefore, allowed rigorous testing of the decoder’s performance under varied biological conditions (e.g., variable-speed inputs and reverse recruitment) as well as post-amputation conditions (e.g., increased biological heterogeneity and changes in cell-type availability). Our results show that a decoder algorithm based on the MN pool spiking rate could be effective in extracting the features of the intended movement and then driving a prosthetic hand with real-time performance (decoding time < 10 ms). Although calibrated by spiking activity resulting from fixed-speed inputs of orderly recruited MNs, the decoder was able to robustly decode the spiking activity of multi-speed inputs, inputs generated from reversed MN recruitment, and inputs reflecting substantial biological heterogeneity of MN properties. This represents proof of concept that this decoder can support long-term fidelity of control system performance with fewer recalibrations, thus enhancing the usability of prostheses for amputees.

### Current Work vs. Earlier MN Decoders

The spiking activity of neurons is mediated via nonlinear voltage-gated and time-dependent ion channels (Hodgkin and Huxley, 1952a, b; Hodgkin et al., 1952). Because of their different activation/inactivation dynamics (i.e., persistent vs. transient currents) and cellular location (i.e., somatic vs. dendritic channels), the activation of these ion channels by synaptic inputs leads to nonlinear changes in the firing activity of MNs (e.g., firing bistability and self-sustained firing behaviors) (Lee and Heckman, 1998, 2000). Accordingly, the relationship between the synaptic input and the MN firing output is not always perfectly linear. While a decoder algorithm based on MN firing activity for prosthetic control has been previously developed and validated using human data (Li et al., 2012, 2013), this algorithm did not consider the nonlinearities in the firing properties of MNs. To capture the nonlinearities in the spiking behavior of MNs and account for them in the decoding process here (i.e., deal with MN firing switching between secondary and tertiary firing behaviors), we employed a neurophysiologically detailed computational model that offered a framework to support the development, optimization, and assessment of the present decoder, which is not feasible in experimental or clinical investigations. The simulations showed that a decoder that captures the initial firing rate as well as estimates the relative change in firing gain between the secondary and tertiary spiking phases could decode the excitation level to MNs accurately. Importantly, these parameters could be easily and accurately estimated from pool or cellular spiking in humans (Farina et al., 2017; Afsharipour et al., 2020; Hassan et al., 2020).

While decoding MN spiking activity for estimating the synaptic input (as a measure of motor intent) or for estimating grip force have been recently examined in animals (Thompson et al., 2018) and humans (Farina et al., 2017; Twardowski et al., 2019; Afsharipour et al., 2020; Hassan et al., 2020), only one decoder in the literature has shown real-time performance for prosthetic control (decoding time < 10 ms, Twardowski et al., 2019). When compared to conventional amplitude-based myoelectric decoders, MN firing activity-based decoders provide more responsive, smooth, and proportional control, supporting the high performance in Twardowski et al. (2019) and even higher performance observed in our results (see Pearson’s CC, RMSE, and NRMSE, next section). However, the decoder developed in Twardowski et al. (2019) is not adaptive, nor tested under conditions of post-amputation complications. Thus, the motor decoder developed in the present study is novel in its adaptability to the changes in motoneuronal spiking activity as well as its adaptability to post-amputation complications. The computational framework used here (clear-box testing with a realistic model of the motor pool) could assist advancement of neural decoders beyond their training datasets by testing and resolving decoder errors resulting from issues of biological heterogeneity and reverse recruitment, which confound consistent performance. This, in turn, could assist development of more responsive decoders which produce motor outputs that more closely reproduce nuanced physiological control.

### Decoder Characteristics

Our results show that our decoder algorithm is accurate, with Pearson’s CC > 0.99 and small NRMSE < 6.3% and RMSE < 3.69). These performance measures exceed those of the Twardowski et al. (2019) decoder. Our results also show that our decoder is computationally efficient in extracting the features of the intended movement and driving a prosthetic hand (i.e., real-time performance with decoding time ∼9 ms). Importantly, the decoder algorithm used the rate of change in spiking rate to estimate the activation speed of the input, then used that information, in real-time, to auto-switch and engage the optimal decoding parameters to decode inputs. This characteristic allows the decoding of a wide range of hand movement speeds (i.e., prosthetic hand opening/closing time ranging between 1 and 12 s) and would be expected to minimize the amputee’s need for frequent decoder calibrations during the day. While the prosthetic hand’s activation speed range was divided in the present study into only three sub-ranges, with each having its own parameter settings, more sub-ranges could be defined and would enhance the decoding accuracy substantially. Additionally, decoding time is expected to substantially improve when run on a dedicated microprocessor in the prosthesis hardware, as the speed testing reported here was conducted on a shared processor of a standard computer.

To rigorously test the developed decoder, we leveraged the “clear-box” advantage of the simulated environment by closely quantifying the decoder’s accuracy under varied conditions likely to be encountered in biological situations, such as the variable-speed input testing, the biological heterogeneity testing, the reverse MN recruitment testing, and the cell-type testing. The variable-speed input evaluation tested the decoder for situations when the activation speed, and consequently the prosthetic hand speed, would be changed in real-time during the movement. Although only calibrated with fixed-speed inputs, but not with variable inputs, the adaptive decoder was able to decode the change in input speed successfully, due to its auto-switching capability. This supports the decoder’s potential in supporting multi-speed movements with speeds varying within its calibration range.

MNs experience changes in their electrical properties, leading to transitions in their types and changes in their ratios of types following peripheral injury and amputation (Titmus and Faber, 1990). This transition process is ongoing, which means a prosthesis decoder must accommodate these changes in MN pool heterogeneity, as well as the higher variability in the pool spiking activity. The heterogeneity tests we conducted in this study, which reflected such variance in MN electrical properties, showed that the decoder, which was not trained with a similar dataset, was still able to decode the MN spiking activity well (NRMSE < 6.55%, RMSE < 3.91, and Pearson’s CC > 0.99). These results indicate that the decoder’s expected performance would continue over the course of these ongoing injury-induced changes in the amputee’s MN pool. As these changes accrue over many months after amputation, the results we present constitute proof of concept that this decoder could significantly reduce the need for recalibration.

The reverse recruitment evaluation tested the decoder’s ability to decode MN spikes generated from a reversed recruitment order (i.e., FF-MNs firing first, followed by FR and eventually S-MNs), despite the decoder being calibrated with only spiking activity resulting from orderly recruited MNs (i.e., S-MNs firing first, followed by FR and eventually FF-MNs—the size principle; Henneman, 1957). Importantly, the decoder successfully decoded the spiking activity of MNs reversely recruited (NRMSE < 13.24%, RMSE < 9.4953, and Pearson’s CC > 0.98). Furthermore, this accuracy could be substantially improved if the decoder were also calibrated with such spiking activity. Because cortical inputs to spinal motoneurons have been shown to favor reverse MN recruitment (Powers et al., 1993; Westcott et al., 1995; Binder et al., 1998), and because mixed and reversed recruitment of MNs have been observed in animals and humans (for review, see Cope and Brian, 1995; Heckman and Enoka, 2012; Bawa et al., 2014), the ability of the decoder to accurately decode the spiking activity resulting from different MN recruitment orders is an important feature.

After amputation, spiking data from remaining motor nerves might be limited to, or dominated by, a given MN cell type. Therefore, we also tested the effect of cell type on the decoder’s performance in decoding the pool spiking. Spiking activity of FR-MNs was the most representative of the full pool activity, probably because of its intermediate properties. This information should guide the selection of spiking data during the calibration process, if the patient’s available MN types can be identified. Altogether, these results support the robustness of the developed decoder in decoding MN spiking activities in a variety of biological situations and thus support the feasibility of this decoder for prosthetic control.

### Study Approach: Strength vs. Limitations

Given that the goal of the present study is to examine the proof of concept of the developed adaptive decoder’s operation and its adaptability to dynamic changes in the amputee’s biological state, our study employed advanced computer simulations to simulate the movement of a prosthetic hand using a “clean” drive approach, which simulates the data with no noise. While the computer simulations allowed testing many scenarios known to emerge after amputation and the “clean” drive approach allowed assessing the basic potential of the developed adaptive decoder, this approach is still limited and ultimately requires human testing. However, this “clean” drive approach is necessary at this proof-of-concept stage because if the decoder fails in a clean testing environment, it would undoubtedly fail with realistic data, and there would be no point in moving forward with further decoder development and human testing. Results of the present study supports the decoder potential. Given the present results, the next step in our investigation will be human testing to test this decoder’s performance under more realistic conditions.

## Conclusion

In conclusion, motor decoders based on the spiking activity of MNs are capable of decoding the firing activity of the MN pool accurately, dynamically, and in real time (<10 ms). The present work supports the proof of concept of such decoders maintaining accurate performance under a number of biological conditions expected to be encountered after amputation. This, in turn, supports the feasibility of this decoder for enabling long-term stability of prosthetic control performance.

## Data Availability Statement

The original contributions presented in the study are publicly available. This data can be found in the figshare data repository at: https://figshare.com/s/e81e92ec3ba50a8dc83a.

## Author Contributions

AM and SE contributed to the conception, design of the study, and wrote the first draft of the manuscript. AM and JA generated the data. AM performed the data analysis. All authors contributed to manuscript revision, read, and approved the submitted version.

## Conflict of Interest

The authors declare that the research was conducted in the absence of any commercial or financial relationships that could be construed as a potential conflict of interest.

## Supplementary Material

Supplementary Video 1 A video recording showing the movement of the MuJoCo prosthetic hand (upper panel) when driven by the adaptive 3-speed decoder in response to a variable input of changing activation speed and direction. The lower panel shows the decoded input (purple) as compared to the actual input (green).
