# Supplementary figures and images for: Adaptive Neural Decoder for Prosthetic Hand Control
Source: Front Neurosci. 2021 Apr 8;15:590775. doi: 10.3389/fnins.2021.590775 (PMC8060566; doi:10.3389/fnins.2021.590775)

## Slide 1
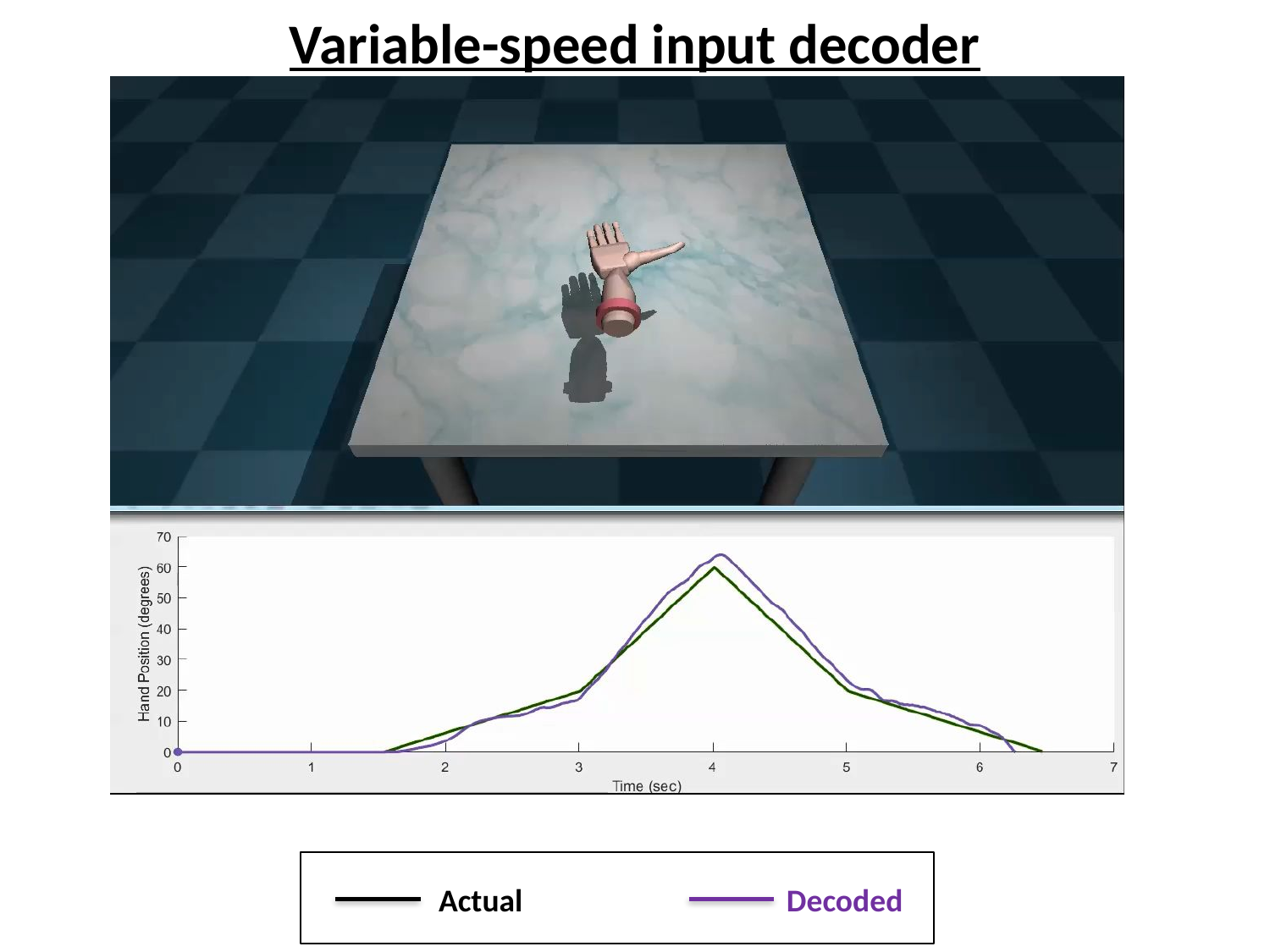

Variable-speed input decoder
Actual
Decoded

Supplement: Supplementary Video 1 — A video recording showing the movement of the MuJoCo prosthetic hand (upper panel) when driven by the adaptive 3-speed decoder in response to a variable input of changing activation speed and direction. The lower panel shows the decoded input (purple) as compared to the actual input (green). [file Presentation_1.PPTX]
